# Tibetan Pig-Derived Probiotic *Lactobacillus amylovorus* SLZX20-1 Improved Intestinal Function *via* Producing Enzymes and Regulating Intestinal Microflora

**DOI:** 10.3389/fnut.2022.846991

**Published:** 2022-03-29

**Authors:** Jiakun Shen, Jie Zhang, Ying Zhao, Zishen Lin, Linbao Ji, Xi Ma

**Affiliations:** ^1^State Key Laboratory of Animal Nutrition, College of Animal Science and Technology, China Agricultural University, Beijing, China; ^2^School of Public Health, North China University of Science and Technology, Hebei, China; ^3^Department of Animal Husbandry and Veterinary, Beijing Vocational College of Agriculture, Beijing, China

**Keywords:** *Lactobacillus amylovorus*, antibacterial activity, acid productivity, adherence ability, gut microbiota composition

## Abstract

The interaction between exogenous microorganisms and the host has received great attention, and finding new probiotics is always the way to improve the health of humans and animals. *Lactobacillus amylovorus* (*L. amylovorus*) is a kind of *Lactobacillus* that can efficiently utilize starch, as a food and feed additive, it has been widely used for mildew prevention and antibacterial, bacteriostasis, and enzyme production. Herein, a strain of *L. amylovorus* was isolated from the feces of Tibetan weaned piglets, named *L. amylovorus* SLZX20-1. Physiological and biochemical experiments *in vitro* confirmed that it had a fast growth rate and could produce a variety of enzymes, including α-galactosidase, β-galactosidase, α-glucosidase, β-glucosidase, and ferulic acid esterase. In addition, *L. amylovorus* SLZX20-1 exerted antibiotic effects on the growth of *Salmonella typhimurium* (*S. typhimurium*) SL1344, *Citrobacter rodentium* (*C. rodentium*) DBS100, *Salmonella pullorum* (*S. pullorum*) CVCC1791, *Staphylococcus aureus* (*S. aureus*) CVCC1882, *Escherichia coli* (*E. coli*) O157, *E. coli* K88, *E. coli* K99, and *E. coli* 987P, which are closely related to acid productivity, such as lactic acid and acetic acid. *In vitro* co-culture, *L. amylovorus* SLZX20-1 has shown the strong adhesion ability to intestinal porcine epithelial cells (IPEC-J2 cells) and activated IPEC-J2 cells with high expression of host defense peptides (HDPs), such as *NK-Lysin, PEP2C*, and *PBD-1*. *In vivo* experiment, *via* intragastric administration, *L. amylovorus* SLZX20-1 significantly improved the feed intake of mice, declined the crypt depth of jejunum and ileum, *L. amylovorus* SLZX20-1 changed the composition of intestinal microbes, especially at the level of colonic genus, the dominant genus was changed from *Lactobacillus* to *S24-7*, which indicated the change of intestinal carbohydrate nutrition. In conclusion, *L. amylovorus* SLZX20-1 showed strong probiotic characteristics, which met with the standard of probiotics and is worth further exploring its impacts on host health and its potential as a candidate strain of probiotics.

## Introduction

The use of probiotics is a promising approach as defencing measures for pathogenic bacteria infections account for their safe characteristics (1). As a potential antibiotic substitute, probiotics are beneficial for host immune function improvement and reduce the occurrence of intestinal diseases in animal husbandry (2). Probiotics can help to prevent or mitigate gastrointestinal infections by stimulating beneficial microorganisms or inhibiting the growth of pathogenic microorganisms, enhancing the barrier function of the intestinal and the local immune system (3, 4). In addition, probiotics play an important role in maintaining the balance of the intestinal environment, affecting the colonization of bacteria in different mucosal parts of the gastrointestinal tract, and the secretion of organic acid, digestive enzymes, and bioactive peptides (5). At present, screening probiotics mainly focus on whether strains have an antibacterial function, and excellent antibiotic alternatives also need to have a certain growth effect, therefore, looking for a variety of biological activities of lactic acid bacteria is very important, such as produce amylase, cellulase, lipase, and protease (6). However, in practice, probiotics often cannot have the above advantages at the same time and the drug resistance and source of probiotics are also important factors that need to be considered in the screening process (7, 8).

*Lactobacillus amylovorus* (*L. amylovorus*) is a probiotic candidate. As a food additive, it has been studied on the antibacterial property (9, 10) and antimildew of bread (11). In addition, it has also been used as an additive in the production of carbonated drinks to reduce obesity (12). In livestock husbandry, *L. amylovorus* is one of the most common silage fermentation additives. It can degrade cellulose and lignin enhanced in silage, increase soluble carbohydrates, sample nutrient storage and utilization, and improve the fermentation quality of silage (13). In addition, in livestock production, *L. amylovorus* has gradually shown excellent probiotic effects, such as increasing daily weight gain (14) and resistance (15). At present, there are a few reports on the safety of *L. amylovorus in vivo* and intestinal microecology.

Tibetan pigs are a herbivorous local pig species with a low incidence during growth. Compared with Landrace pigs, the intestinal flora of Tibetan pigs is more abundant, and fiber degrading bacteria are rich in the large intestine, which can decompose cellulose and other substances in crude feed to facilitate host digestion and absorption of nutrients (16). Moreover, Tibetan pigs are hardly fed or injected with antibiotics during the feeding process, and the probability of drug-resistant strains is very low, so it has great potential to isolate potential probiotics from Tibetan pigs. *L. amylovorus* are characteristic bacteria in piglets, from day 1 to day 11 after weaning, these bacteria are the dominant species in the small intestine (17), which are easy to isolate and culture. The current research focuses on the main physiological and biochemical characteristics, antibacterial activity, and adhesion ability of *L. amylovorus* SLZX20-1 isolated from Tibetan pigs. The effects of intestinal tissue morphology and intestinal microbial composition are determined through *in vivo* experiments to verify the probiotic function and safety of the isolated *L. amylovorus* SLZX20-1.

## Materials and Methods

### Isolation, Purification, and Identification of *L. amylovorus* SLZX20-1 Strains

Feces were collected from a pig farm located in south Tibetan and were moved back to the lab as soon as possible. Then mixed with 70% glycerol as a 1:1 ratio to be fecal samples and stored at −80°C. When it is needed, 1 g of fecal samples was mixed with normal saline *via* vortex oscillations to get suspensions. Suspensions were diluted in a 10-fold gradient and the serial dilutions were streaked onto selective medium, anaerobic incubated (Anaerobic Incubator LAI-D2, Longyue, Shanghai, China) at 37°C till a single colony of appropriate size was grown. Picked single colony was inoculated on de Man, Rogosa, and Sharpe (MRS) solid medium plates in anaerobic conditions at 37°C to purify them and repeated this process three times for further purification. Purified strains were anaerobically incubated in MRS broth for 18 h. Then mixed bacterial suspension with 50% glycerol as 1:1 ratio and cryopreserved at −80°C for further identification.

The colonies were observed by size, shape, color, margins, and pellucidity. The isolates were done the Gram staining and were observed under an oil microscope (1,000 ×) to identify morphology for further classifying the strains. The utilization of starch by strains was detected by culturing in starch medium, and the starch in the medium was detected by iodine solution. Total DNA of purified strains was extracted using bacteria genomic DNA kit (CWBIO, Beijing, China). Molecular identification was done by PCR amplification using 27F and 1492R universal primers (Weisburg WG, 1991) with 1,500-bp product size. The forward primer is 5′-AGAGTTTGATCCTGGCTCAG-3′ and the reverse primer is 5′-GGTTACCTTGTTACGACTT-3′. PCR reactions were conducted in 50 μl reactions that include 1 μl of template DNA, 25 μl of 2.5 mM deoxynucleotide triphosphates (dNTPs), 22 μl of deionized water, 1 μl of 10 μM primer for three replications. For initial denaturation at 94°C for 4 min, then at 94°C for 45 s by 30 cycles, 55°C for 45 s, and 72°C for 1 min and an extension at 72°C for 10 min. The product of PCR was sequenced and blasted with the National Center for Biotechnology Information (NCBI) database. High homological *Lactobacillus* sequences of different species and different strains were selected to contrast with the sequences of the isolates to the construction of phylogenetic tree *via* Mega 7.0 software. The identified isolates were named *L. amylovorus* SLZX20-1 and kept in China General Microbiological Culture Collection Center (strain No. 20122).

### Growth Characteristics, pH and Bile Salts Tolerance

Streaked and inoculated *L. amylovorus* SLZX20-1 onto a plate to activate the strain. In total, 1% of activated strains was anaerobically incubated in MRS broth at 37°C and tested OD600 nm value, pH, and counted viable bacteria numbers every 2 h. The *L. amylovorus* suspensions were diluted in a 10-fold gradient and streaked onto MRS medium, anaerobic incubated at 37°C to count viable bacteria numbers in plates, which colony numbers were between 30 and 300.

Tolerance to low pH and bile salts were assessed that 1% of activated strains was incubated with different pH (0, 2.5, 3.0, and 4.0) of sterile phosphate buffered saline (PBS) or with sterile PBS (Gibco, Brooklyn, NY, USA) containing different bile salts (Solarbio, Beijing, China) levels (0, 0.1, 0.2, and 0.3%). The mixture was incubated at 37°C for 0, 1, 2, 3, and 4 h to count viable bacteria numbers *via* the flat colony counting method. Taking the viable bacteria number of 0 h as the control, the survival rate of *L. amylovorus* SLZX20-1 in different pH conditions or different bile salt levels was calculated.

### Antibiotic Sensitivity Assay

Antibiotic sensitivity was determined by drug-sensitive paper tablets (Hangzhou microbial reagent Co. LTD, China). Selected 1–2 common antibiotics from each class as a representative to comprehensively reflect the drug sensitivity of *L. amylovorus* SLZX20-1, such as amikacin, gentamicin, cefradine, carbenicillin, penicillin, norfloxacin, tetracycline, chloramphenicol, vancomycin, and erythromycin. In total, 1% of *L. amylovorus* SLZX20-1 was mixed in melted MRS solid medium till it solidified and dried for 3–5 min. The tablets were homogenized, spread in the plate, and gently compacted, with a spacing between each other not less than 24 mm. The distance from tablet center to the edge of plate was not less than 15 mm which prevents the crossing between each transparent circle. Plates were incubated in a 37°C anaerobic incubator for 36 h to observe the size of antibacterial circles. Every tablet was repeated in triplicate. The circle diameter <15 mm was resistant and 16–20 mm for medium sensitivity, the circle diameter of >20 mm was sensitive.

### Enzymatic-Production Capacity

The determination of enzymatic spectrometry was done by using API-ZYM kit (Biomérieux, France). After 14 h cultures, *L. amylovorus* SLZX20-1 was centrifuged at 5,000 rpm for 10 min, removed the supernatant, resuspended the strains *via* normal saline, and adjusted their concentrations at 1 × 10^9^ colony forming unit (CFU)/ml. In total, 65 μl of bacteria suspension was added into strip wells and co-cultured in the anaerobic incubator at 37°C for 4 h. Then added a color developer into wells and recorded the color reaction results.

The supernatant of *L. amylovorus* SLZX20-1 was filtered and added into solid mediums containing ethyl4-hydroxy-3-methoxycinnamate to incubate at 37°C for 24 h for qualitative analysis of the feruloyl esterase production *via* the Oxford Cup method. Quantitative determination of feruloyl esterase activity was done by hyphenated to liquid chromatography (HPLC) (Agilent Technologies 1200 Series, Santa Clara, CA, USA) separation systems. The enzyme activity was defined that the required enzyme amount for degradation of ethyl4-hydroxy-3-methoxycinnamate per minute to generate 1 μmol ferulic acid is 1 U as the condition of 39°C, pH 7.0. After 36 h cultures, *L. amylovorus* SLZX20-1 was centrifuged at 5,000 rpm for 10 min, discarded the supernatant, washed strains, resuspended it with PBS, and then was broken by ultrasonic to obtain a crude solution of feruloyl esterase. In total, 400 μl crude solution of feruloyl esterase, 100 μl 10 mmol/l ethyl4-hydroxy-3-methoxycinnamate (solute with dimethyl sulfoxide), 500 μl sodium phosphate buffer of contained 2.5% (V/V) Triton-100 (pH 7.0) were co-incubator at 39°C for 45 min, then heated in 100°C for 10 min to terminate the reaction. Detected the production amount of ferulic acid *via* HPLC separation systems.

### Antimicrobial Activity and Antimicrobial Substances Assay

Antimicrobial activity was assessed against eight pathogens associated with *Escherichia coli (E. coli)* O157, K88, K99, 987P, *Salmonella typhimurium (S. typhimurium)* SL1344, *Citrobacter rodentium (C. rodentium)* DBS100, *Salmonella pullorum (S. pullorum)* CVCC1791, and *Staphylococcus aureus (S. aureus)* CVCC1882 (all strains were obtained from China Veterinary Culture Collection Center). Oxford Cup test was done for the detection of antimicrobial activity. Antibiotics (200 μl 0.1 mg/ml doxycycline hydrochloride) were used as a positive control, normal saline was used as a negative control. Briefly, *L. amylovorus* SLZX20-1 36 h cultures were centrifuged (5,000 × g for 10 min, 4°C), the supernatant and the strains were collected separately. The supernatant (filter by 0.22 μm filters), strains (wash three times and resuspend with sterile normal saline), and suspensions (*L. amylovorus* SLZX20-1 36 h cultures without centrifuging) were set to be three treatment groups. A drop of 200 μl antibiotics, normal saline, supernatant, strains, or suspensions was independently added into each hole of medium and co-cultured with different pathogens in the incubator for 12 h. Then the diameter of the inhibition zone was measured. Every group was repeated in triplicate.

Collected supernatant of *L. amylovorus* SLZX20-1 36 h cultures via centrifugation (5,000× g for 10 min, 4°C) and filtered it. In the exploration of antimicrobial substances, supernatant without any modification (pH is 4) was set as a control, adjusted pH of supernatant to 7, added protease k (1 mg/ml, adjusted pH to 8), trypsin (1 mg/ml, adjust pH to 3), or pepsin (1 mg/ml, adjust pH to 8.2) in the supernatant, which these treatments were constituted to be trial groups. Stood protease k, trypsin, and pepsin groups at 37°C for 2 h and then adjusted their pH to 7. A drop of 200 μl supernatant from every group was independently added into each hole of medium and co-cultured with different pathogens in the incubator at 37°C for 24 h. Then measured the diameter of the inhibition zone. Furthermore, the short-chain fatty acid (SCFA) production of the supernatant was analyzed by ion chromatography after a 400-fold dilution. SCFA was measured according to our previous studies (18, 19).

### Adherence to Intestinal Porcine Epithelial Cells (IPEC-J2 cells)

Recovery and passage of IPEC-J2 cells were referred to using the same method previous study (20). Before IPEC-J2 cells were fused to 90, 1% *L. amylovorus* SLZX20-1 was inoculated to MRS broth medium and grown for 12–14 h. Then, the mixture was centrifuged at 4°C 5,000 rpm for 10 min. The supernatant was discarded, and the strains were washed three times with PBS and resuspended by Dulbecco's Modified Eagle-F12 Ham Medium (DMEM/F12, Gibco, NY, USA) without serum and antibiotics and adjusted concentrations at 1 × 10^7^, 1 × 10^8^, or 1 × 10^9^ CFU/ml. Adhesion rate was determined by co-culture of *L. amylovorus* SLZX20-1 and IPEC-J2 cells. Different concentrations of *L. amylovorus* SLZX20-1 were added to six-well plates with grown and without non-adherent cells. They were incubated in an atmosphere of 5% CO_2_ incubator at 37°C for 2 h. Then, discarded the supernatant and washed three times with PBS to fully remove the unadhered bacteria. In total, 1 ml of 0.1% Triton-100 (Sigma, Saint Louis, MO, USA) was added to each well, standing for 15 min to fully lyse the cells, free the bacteria, and then the bacterial fluid was moved into a centrifuge tube. The adhesion rate of the *L. amylovorus* SLZX20-1 was calculated *via* counting the numbers of live bacteria before and after adhesion by the flat colony counting method. Moreover, gram staining was used to observe the adhesion ability. Specifically, the process was similar to methods mentioned above except for the following points. Firstly, the IPEC-J2 cells were climbed on the glass slide. Secondly, after washing the unadhered bacteria, the cells and adhesive bacteria were fixed via formaldehyde for 30 min. Following, gram staining was done and the cell slides were put on the glass slides. Finally, adhesion condition of the co-culture of *L. amylovorus* SLZX20-1 and IPEC-J2 cells were examined under a microscope.

### Suppress Pathogen Adhesion to IPEC-J2 Cells

Separately cultured *L. amylovorus* SLZX20-1 and *Enterotoxigenic Escherichia coli* (ETEC) K88, collected strains and washed three times with PBS, resuspended by DMEM/F12, and adjusted concentrations of ETEC K88 at 1 × 10^7^ and 1 × 10^8^ CFU/ml. Competition, exclusion, and replacement trials were performed to test the antibacterial activity of *L. amylovorus* SLZX20-1. Co-culture of *L. amylovorus* SLZX20-1 and ETEC K88 onto IPEC-J2 cell plate for 2 h to test their competition. *L. amylovorus* SLZX20-1 was added onto the IPEC-J2 cell plate and incubated in an atmosphere of 5% CO_2_ incubator at 37°C for 1 h. Then the supernatant was discarded and plates were washed three times with sterile PBS, subsequently, ETEC K88 was added to co-culture for 1 h again to test their repellency. The processes of the replacement trial were the same as the exclusion trial, but the difference between them lies in a different order of *L. amylovorus* SLZX20-1 addition. To be specific, ETEC K88 and IPEC-J2 cells were cocultured for 1 h, and *L. amylovorus* SLZX20-1 were add to the mixture for another 1 h. In three trials, all the controls were using DMEM/F12 to replace *L. amylovorus* addition. Finally, the inhibition rate of the *L. amylovorus* SLZX20-1 on ETEC was calculated *via* the count of the numbers of live ETEC K88 by the flat colony counting method. The formula of inhibition rate equal to the difference between the controls minus the experimental group was divided by the control group.

### Expression of Host Defense Peptide (HDPs) on IPEC-J2 Cells

Intestinal porcine epithelial cells were added to six-well plates and grew till 80% of cells were confluent. After 14 h cultures, *L. amylovorus* SLZX20-1 were centrifuged, discarded the supernatant, washed strains with normal saline, resuspended by DMEM/F12 without serum, and set low, medium, and high-dose groups, in which bacteria suspension concentrations are 1 × 10^7^, 1 × 10^8^, and 1 × 10^9^ CFU/ml. In total, 2 ml different concentrations of *L. amylovorus* SLZX20-1 were added to six-well plates of IPEC-J2 cells, co-incubated in an atmosphere of 5% CO_2_ incubator at 37°C for 6 h. RNA extraction was performed according to the instruction of cell RNA extraction kit (CWBIO, Beijing, China) and RNA reverse transcription**-**PCR (RT**-**PCR) was performed according to the instruction of RNA reverse transcription kit (Mei5 Biotechnology, Beijing, China) and done by qRT**-**PCR instrument (LightCycler 96, Roche, Shanghai, China). The primer sequence design of six HDPs is shown in Supplementary Table 1. The reaction system of real-time fluorescent quantitative PCR reactions was conducted in 10 μl reactions that include 2 μl of template DNA, 2 μl of real-time PCR super mix, 2 μl of deionized water, 0.5 μl of 10 μM forward primer, and 0.5 μl of 10 μM reverse primer. For initial denaturation at 95°C for 45 s, 95°C for 15 s then at 60°C for 15 s by 35 cycles, an extension at 72°C for 45 s.

### Animal Experimental Protocol

All the procedures of this experiment were approved by the animal protection and utilization organization committee of China Agricultural University (CAU20171015-3). Sixty weaning C57BL/6 male mice were randomly divided into four groups, which are control group (CON, 150 μl normal saline), low dose group (LOW, 1 × 10^7^ CFU/ml), medium dose group (MID, 1 × 10^8^ CFU/ml), and high dose group (HIGH, 1 × 10^9^ CFU/ml) for intragastric administration of *L. amylovorus* SLZX20-1. Each group has three replicates, every replicate has five mice. Intragastric administration was performed every 2 days and the trial period is 14 d. Daily survival and abnormal status of mice were observed and recorded. Daily weight and feed intake were recorded too. Six mice were randomly selected from each group for sampling on day 14. Based on the daily weight changes and feed intake to calculate the average daily weight gain (ADG), average daily intake (ADFI), and feed to gain ratio (ratio of ADFI to ADG, F:G). Separated jejunum, ileum, cecum, colon, and collected chyme inside in 1.5 ml sterile centrifuge tube, put in liquid nitrogen immediately, and stored at −80°C for subsequent 16S rDNA sequencing.

### Illumina MiSeq Sequencing

After purification with AxyPrep DNA Purification kit (Axygen Biosciences, Union City, CA, USA), the PCR products were detected by agarose gel (2%) electrophoresis and were quantified using PicoGreen dsDNA Quantitation Reagent (Invitrogen, Waltham, MA, USA) on QuantiFluor-ST Fluorometer (Promega, Madison, WI, USA). After that, according to standard protocols, collected amplicons for paired-end sequencing (2 × 300 bp). This process did on the Illumina MiSeq platform (Allwegene, China). The raw data of this manuscript have been uploaded to NCBI SRA database, and the accession No. is PRJNA792839.

### Bioinformatics Analysis of Sequencing Data

For raw FASTQ files analysis, the first step was demultiplexed and quality-filtered *via* QIIME (version 1.17). It follows with some basic principles: (i) sequencing reads were trimmed at the sites with an average quality score <20 over a 50 bp's sliding window and deleted trimmed reads <50 bp; (ii) the reads that contained mismatching barcode were deleted; and (iii) removed the paired reads with <10 bp overlapping.

UPARSE (version 7.1, http://drive5.com/uparse/) was used to gather OTUs with a 97% similarity. For chimeric sequences, using UCHIME identified and deleted. RDP Classifier (http://rdp.cme.msu.edu/) based on Silva (SSU115) 16S rRNA database was used to do the taxonomic analysis for each 16S rRNA gene sequence, and the confidence threshold is 70%. The diversity indexes, including Chao index, Shannon index, coverage indexes, and Metastats analysis, all were dependent on procedure Mothur v.1.21.1 to conduct. Primer 6 software (Primer-E Ltd., UK) was used for hierarchical clustering analysis. Software Venn diagrams for Venn figures and R tools for bacterial community figures. Using R tools to analyze the PCoA analysis. The significant differences between the two groups of microbial types were analyzed by linear discriminant analysis Effect Size (LEfSe) analysis.

### Statistical Analysis

The data and graphic analysis were performed by GraphPad prism 8.0. The results were shown on mean ± SEM. *p* < 0.05 was considered that differences are significant.

## Results

### Identification of *L. amylovorus* SLZX20-1

**Figure 1:**
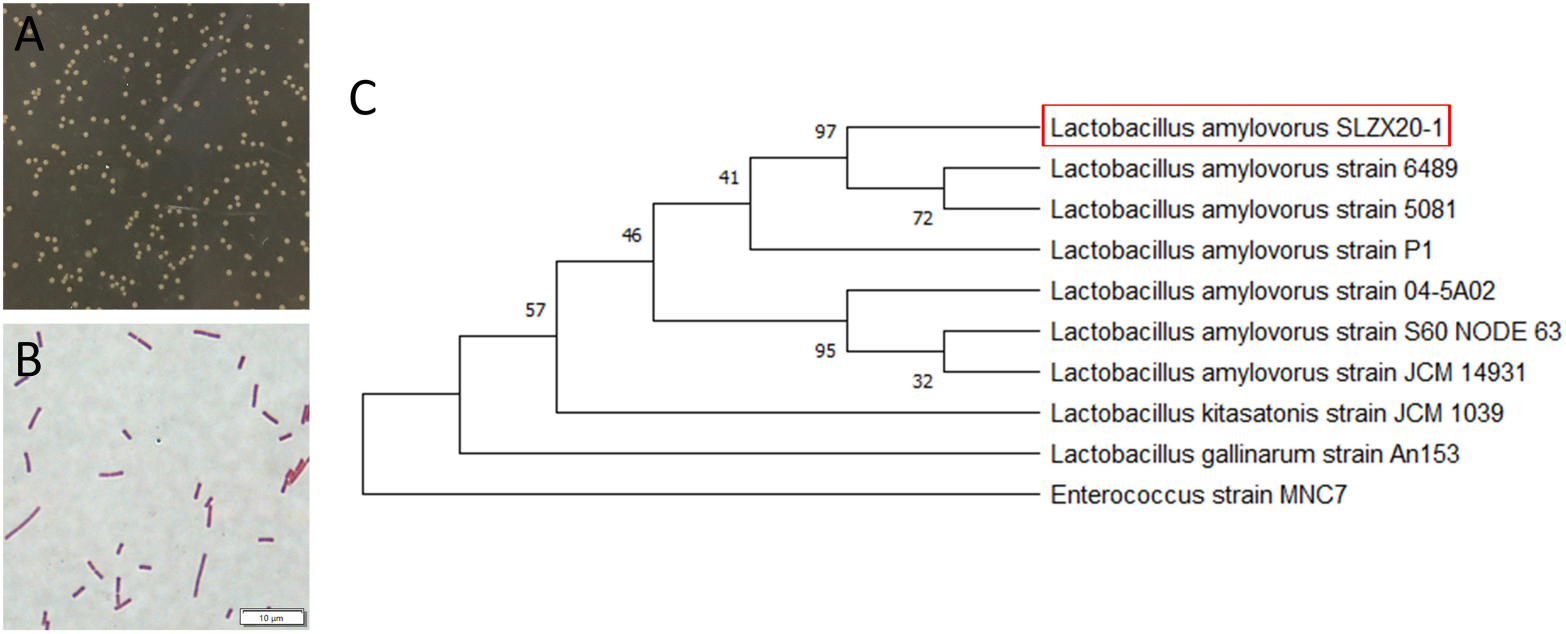
Identification of *L. amylovorus* SLZX20-1. **(A)** Colony morphology of *L. amylovorus* SLZX20-1. **(B)** Gram staining of *L. amylovorus* SLZX20-1, bar = 10 μm. **(C)** The phylogenetic tree of *L. amylovorus* SLZX20-1.

The morphology of colonies is shown in Figure 1A that diameter was about 1–2 mm with milky white color, opaque, smooth surface, and neat edges. Strains of *L. amylovorus* SLZX20-1 were proven to be the Gram-positive bacteria with rod-shaped, about 2–5 μm (Figure 1B). The amplified products size of 16S rDNA was about 1,500 bp and the sequencing length was 1,464 bp (Supplementary Figure 1). Sequencing results of 16S rDNA sequence were submitted to NCBI database for Basic Local Alignment Search Tool (BLAST) detection and were analyzed consanguinity *via* phylogenetic tree, which showed that the strains had 99.66% homology with *L. amylovorus* and genetically related with to *L. amylovorus* (Figure 1C). Further, *L. amylovorus* are major starch utilizers, we observed the utilization of starch by the isolated strain in starch medium (Supplementary Figure 2). Therefore, the strain was named as *L. amylovorus* SLZX20-1.

### Probiotic Properties of *L. amylovorus* SLZX20-1 *in vitro*

**Figure 2:**
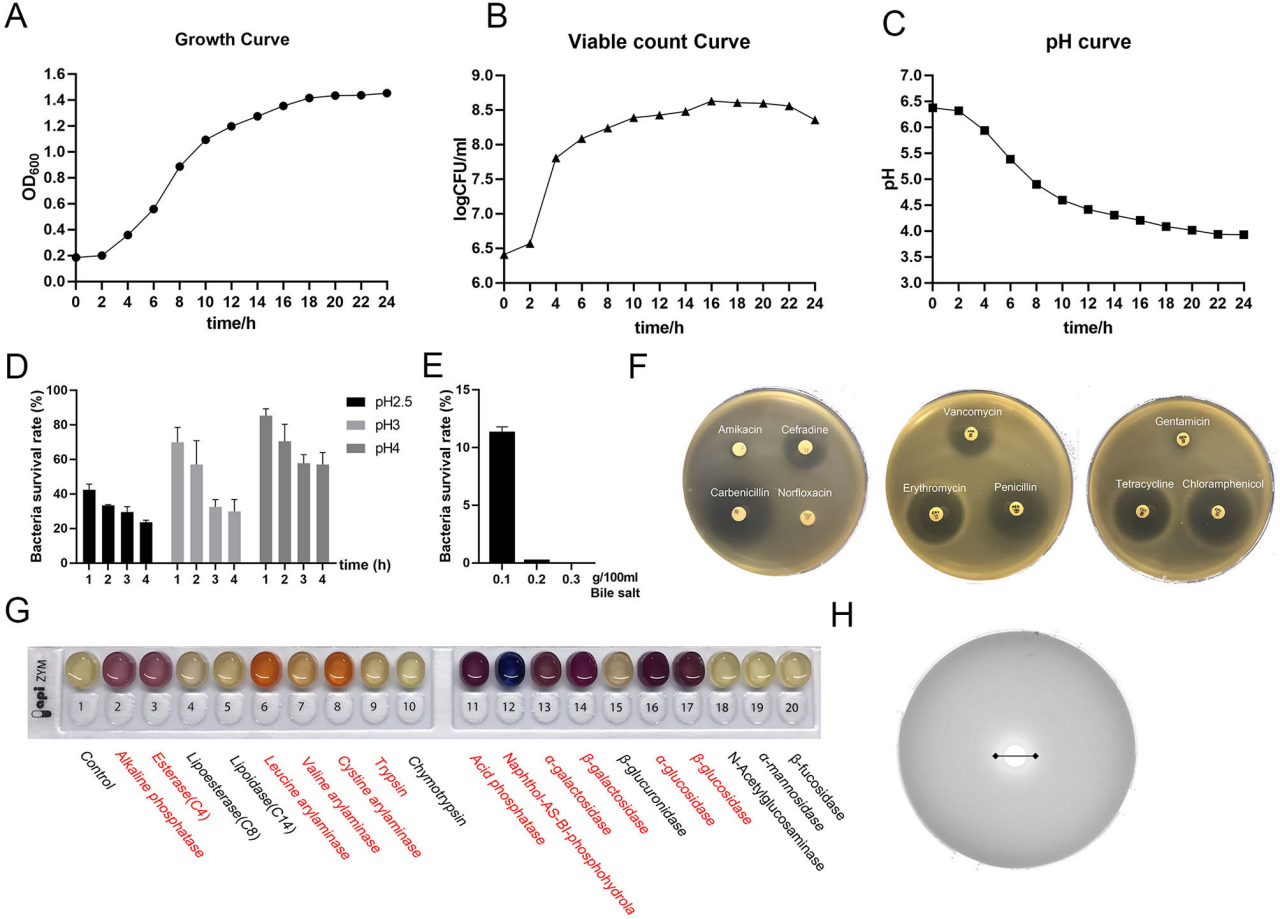
Probiotic properties of *L. amylovorus* SLZX20-1. **(A–C)** The growth curve, viable count curve, and pH curve of *L. amylovorus* SLZX20-1. **(D)** The ability of acid tolerance of *L. amylovorus* SLZX20-1. **(E)** The ability of bile salt tolerance of *L. amylovorus* SLZX20-1. **(F)** The antibiotic sensitivity of *L. amylovorus* SLZX20-1. **(G)** API-ZYM enzyme activities assay of *L. amylovorus* SLZX20-1, enzyme marked in red represents positive reaction. **(H)** The ability of feruloyl esterase production of *L. amylovorus* SLZX20-1.

The growth stability period of *L. amylovorus* SLZX20-1 indicated that the strains grew rapidly (Figure 2A) and multiplied in nutrient-rich environments in a short time (Figure 2B). The number of live bacteria reached 1 × 10^8^ CFU/ml in 6 h and maintained this number till 24 h. When the strains entered the log growth period, the fluid pH of strains decreased quickly and slowed the decline rate bounded after 12 h with pH 3.93 at 24 h (Figure 2C), indicating the strong acid production capacity of *L. amylovorus* SLZX20-1. Meanwhile, *L. amylovorus* SLZX20-1 has shown strong acid tolerance ability at pH 4 and pH 3 with 70.55 and 57.13% survival rate for 2 h (Figure 2D). In addition it can tolerate lower concentration (0.1%) of bile salt environment instead of higher concentration (0.2%) of bile salt environment (Figure 2E). *L. amylovorus* SLZX20-1 was sensitive for cefradine, carboxypenicillins, tetracycline, chloramphenicol, vancomycin, erythromycin, and penicillin but insensitive to butamkana, norfloxacin, and gentamicin (Figure 2F). It can utilize fibrodilose, maltose, salicin, sucrose, raffinose, lactose, and synanthrin (shown in Supplementary Table 2). *L. amylovorus* SLZX20-1 has the stronger ability for enzyme production on leucine aromaminase, cystine amminoaraminase, acid phosphatase, Naphthol-AS-BI-phosphohydrolase, α-galactosidase, β-galactosidase, α-glucosidase, and β-glucosidase, while weaker on alkaline phosphatase, esterase, valine aromaminase and trypsin (Figure 2G; Supplementary Table 3). Meanwhile, *L. amylovorus* SLZX20-1 proved that the strains can secrete feruloyl esterase to degrade ethyl ferulate. In addition, the viability unit of feruloyl esterase was 105.7447 mU/mgprot *via* 72 h culture and liquid chromatography quantification analysis (Figure 2H).

### Drug Resistance and Antibacterial Activity *L. amylovorus* SLZX20-1

**Figure 3:**
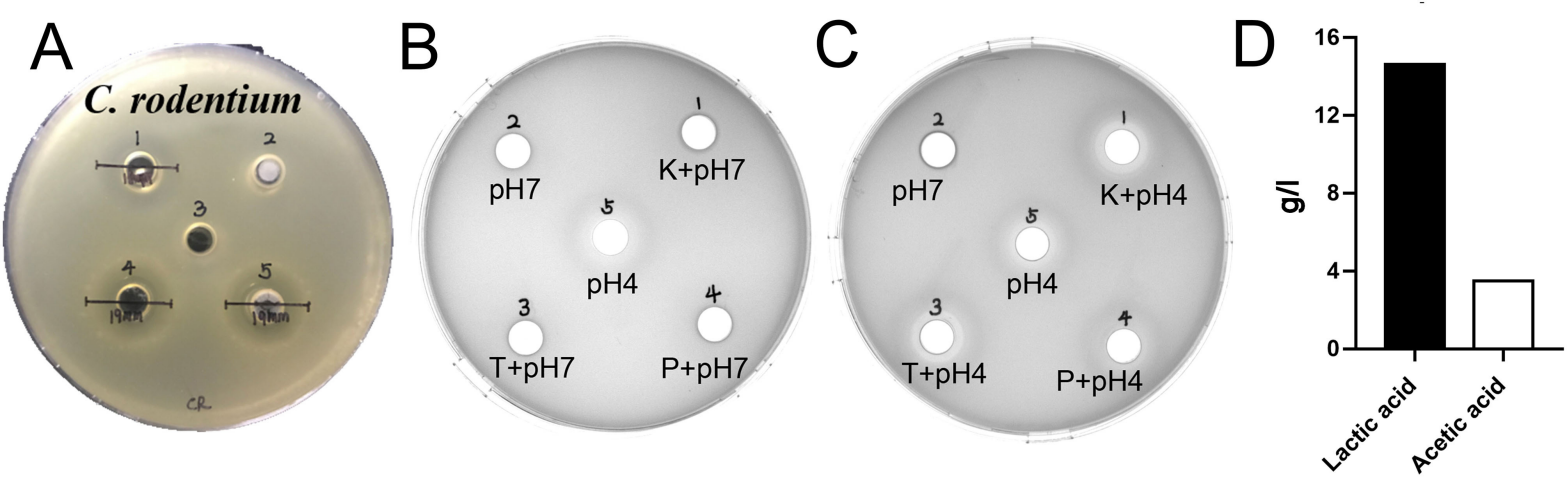
Antibacterial activity and acid production capacity of *L. amylovorus* SLZX20-1. **(A)** The inhibitory effect of *L. amylovorus* SLZX20-1 against *C. rodentium* DBS100, Hole 1 is the positive control group (doxycycline), hole 2 is the group of sediment of *L. amylovorus* SLZX20-1, hole 3 is the negative control group, hole 4 is a group of the fermentation broth of *L. amylovorus* SLZX20-1, hole 5 is a group of bacterial suspension of *L. amylovorus* SLZX20-1. **(B,C)** The exploration of antibacterial substances of *L. amylovorus* SLZX20-1, K-Protease K, T-Trypsin, P-Pepsin. **(D)** The concentration of main short-chain fatty acids produced by *L. amylovorus* SLZX20-1.

The results of antimicrobial activity detection have shown that *L. amylovorus* SLZX20-1 can inhibit the growth of *S. typhimurium* SL1344, *C. rodentium* DBS100, *S. pullorum* CVCC1791, *S. aureus* CVCC1882, *E. coli* O157, *E. coli* K88, *E. coli* K99, and *E. coli* 987P, for which the inhibition ability for *C. rodentium* DBS100 was stronger than others (Figure 3A, Supplementary Figure 3, Table 1). *C. rodentium* DBS100 was further used to explore the antibacterial reagent in *L. amylovorus* SLZX20-1, the result showed that adjusted pH to neutral dismissing the antimicrobial activity of *L. amylovorus* SLZX20-1, which indicated that the inhibition effect was mainly exerted by the low pH of supernatant, due to its strong acid-producing capacity rather than proteins or peptides production from *L. amylovorus* SLZX20-1 (Figures 3B,C). The SCFAs in their supernatant were analyzed by ion chromatography. It was found that lactic acid and acetic acid were mainly produced. The yields of lactic acid and acetic acid were 14.62 and 3.51 g/l, respectively (Figure 3D).

**Table 1:** The diameter of the inhibition zone of *L. amylovorus* SLZX20-1 against pathogenic bacteria.

**Indicator species**	**Inhibitory zone (mm)**			
	**Fermentation broth**	**Suspension**	**Sediment**	**Doxycycline**
*E. coli* K88	15.95 ± 0.87	15.75 ± 0.03	–	–
*E. coli* K99	16.03 ± 0.26	16.13 ± 0.61	–	17.40 ± 0.29
*E. coli* O157	15.80 ± 0.53	15.67 ± 0.68	–	12.90 ± 0.26
*E. coli* 987P	16.53 ± 0.39	15.87 ± 0.53	–	–
*S. typhimurium* SL1344	17.63 ± 0.30	17.00 ± 0.08	–	17.43 ± 0.30
*C. rodentium* DBS100	18.47 ± 0.03	18.37 ± 0.19	–	17.53 ± 0.19
*S. pullorum* CVCC 1791	17.6 ± 0.29	17.53 ± 0.19	–	19.97 ± 0.75
*S. aureus* CVCC1882	17.07 ± 0.54	16.57 ± 0.56	–	28.17 ± 0.73

### Adhesion of *L. amylovorus* SLZX20-1 to IPEC-J2 cells

**Figure 4:**
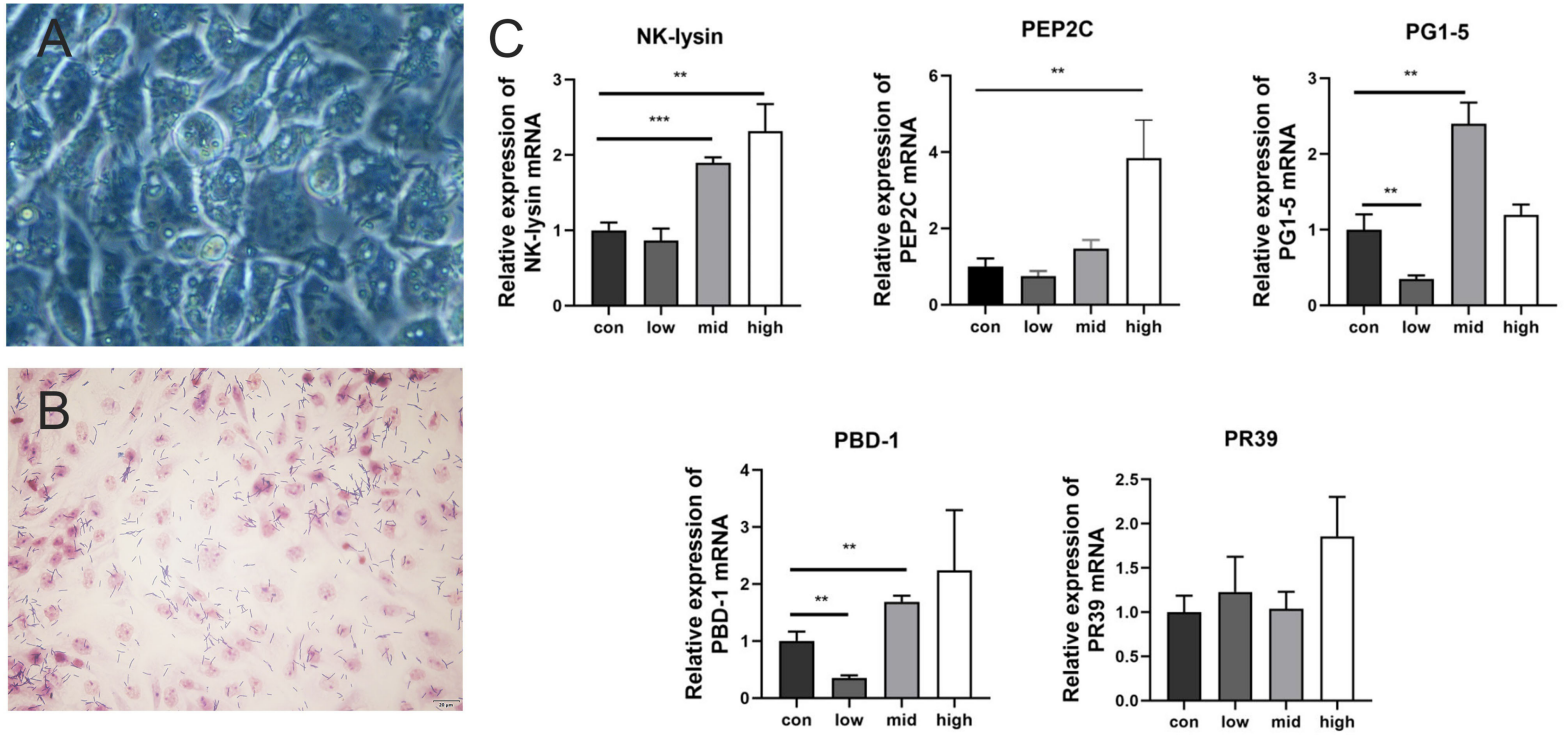
Adhesion of *L. amylovorus* SLZX20-1 to IPEC-J2 cells. **(A)** The co-culture of *L. amylovorus* SLZX20-1 and IPEC-J2 cells after being washed with PBS was observed under an inverted microscope, 200×. **(B)** The gram staining of *L. amylovorus* SLZX20-1 co-cultured with IPEC-J2 cells, bar = 20 μm. **(C)** The effect of different doses of *L. amylovorus* SLZX20-1 on host defense peptides expression of IPEC-J2 cells. **p < 0.01, ***p < 0.001, compared to the con.

*L. amylovorus* SLZX20-1 had certain adhesion for IPEC-J2 cells and this process was not easily affected by physical stimulation, such as washing and tinting (Figures 4A,B). Adhesion rates were 26.69, 20.21, and 4.38% by co-culturing with IPEC-J2 cells at low, medium, and high dose of *L. amylovorus* SLZX20-1. These results have shown that the adhesive rate of *L. amylovorus* SLZX20-1 was decreased following the increasing concentration of *L. amylovorus* SLZX20-1 per unit area of IPEC-J2 cells (Table 2). However, *L. amylovorus* SLZX20-1 proved that it did not inhibit *E. coli* K88 adhering to IPEC-J2 cells in every competition, exclusion, and replacement test (Supplementary Table 4). *L. amylovorus* SLZX20-1 was co-cultured with IPEC-J2 cells that significantly improved the expression level of antimicrobial peptides mRNA for *NK-lysin, PEP2C, PG1-5*, and *PBD-1*. Among that, high dose significantly improved *NK-lysin, PEP2C* expression (*p* < 0.05), medium dose significantly improved *NK-lysin, PG1-5, PBD-1* expression (*p* < 0.05), but low dose significantly decreased *PG1-5* expression (*p* < 0.05; Figure 4C).

**Table 2:** The adhesive capacity of different doses of *L. amylovorus* SLZX20-1 to IPEC-J2.

**Items**	**Low dose**	**Middle dose**	**High dose**
CFU before the adhesion	1.60 × 10^7^	1.10 × 10^8^	8.60 × 10^8^
CFU after the adhesion	4.43 × 10^6^	1.49 × 10^7^	3.77 × 10^7^
Adhesion rate (%)	26.69	20.21	4.38

### The Influence of Feed-supplementation With *L. amylovorus* SLZX20-1 on the Intestinal Microenvironment of Mice *in vivo*

Intragastric administration of different doses of *L. amylovorus* SLZX20-1 had no significant effect on the weight change of mice but showed a certain effect on promoting weight gain. There was no significant difference in the final body weight, ADG, and F:G ratio among the treatment groups (*p* > 0.05), but the ADF intake of the high group increased significantly (*p* < 0.05; Table 3).

**Table 3:** Effects of different doses of *L. amylovorus* SLZX20-1 on growth performance in mice.

**Items**	**CON**	**LOW**	**MID**	**HIGH**	**SEM**	***P*** **value**
Initial weight (g)	12.88	13.21	12.96	13.05	0.07	0.40
Final weight (g)	19.73	20.39	20.05	20.39	0.17	0.43
ADG (g/d)	0.53	0.55	0.55	0.56	0.01	0.72
ADFI (g/d)	2.78^a^	2.87^ab^	2.93^ab^	2.95^b^	0.03	0.11
F:G	5.27	5.21	5.40	5.27	0.08	0.91

*CON, 150 μl normal saline; LOW, 150 μl 1 × 10^7^ CFU/ml L. amylovorus SLZX20-1; MID, 1 × 10^8^ CFU/ml L. amylovorus SLZX20-1; HIGH, 1 × 10^9^ CFU/ml L. amylovorus SLZX20-1. SEM means standard error of the mean, different letter superscripts in the same row indicate a significant difference (p < 0.05), n = 6*.

The tissue morphology figures of the jejunum, ileum, and colon of mice are shown in Supplementary Figure 4. The results show that the tissue structure of each intestinal segment *L. amylovorus* SLZX20-1 mice with different doses by gavage was clear, and the intestinal villus of the small intestine were closely arranged. By measuring the villus height and recess depth of jejunum and ileum, it was found that different doses of *L. amylovorus* SLZX20-1 by gavage significantly reduced the recess depth of jejunum and ileum (*p* < 0.05). In addition, the villus height of mice in the low group was significantly reduced (*p* < 0.05), and the villus ratio of jejunum and ileum was significantly increased in the high dose group (*p* < 0.05; Table 4).

**Table 4:** Effect of *L. amylovorus* SLZX20-1 on intestine development in mice.

**Items**	**CON**	**LOW**	**MID**	**HIGH**	**SEM**	***P*** **value**
Jejunum						
Villus height (μm)	464.37	452.10	430.63	483.44	13.70	0.580
Crypt depth (μm)	135.61^a^	121.30^b^	113.09^b^	117.58^b^	2.38	0.001
V/C	3.42^a^	3.73^ab^	3.81^ab^	4.10^b^	0.10	0.132
Ileum						
Villus height (μm)	291.21^b^	245.66^a^	290.64^b^	312.69^b^	6.99	0.002
Crypt depth (μm)	124.24^a^	108.84^b^	109.87^b^	108.16^b^	2.01	0.001
V/C	2.34^a^	2.26^a^	2.64^b^	2.89^b^	0.07	0.001

*CON, 150 μl normal saline; LOW, 150 μl 1 × 10^7^ CFU/ml L. amylovorus SLZX20-1; MID, 1 × 10^8^ CFU/ml L. amylovorus SLZX20-1; HIGH, 1 × 10^9^ CFU/ml L. amylovorus SLZX20-1*.

*SEM means standard error of the mean, different letter superscripts in the same row indicate a significant difference (p < 0.05), n = 6*.

### Effects of *L. amylovorus* SLZX20-1 Microbial Composition of the Ileum

**Figure 5:**
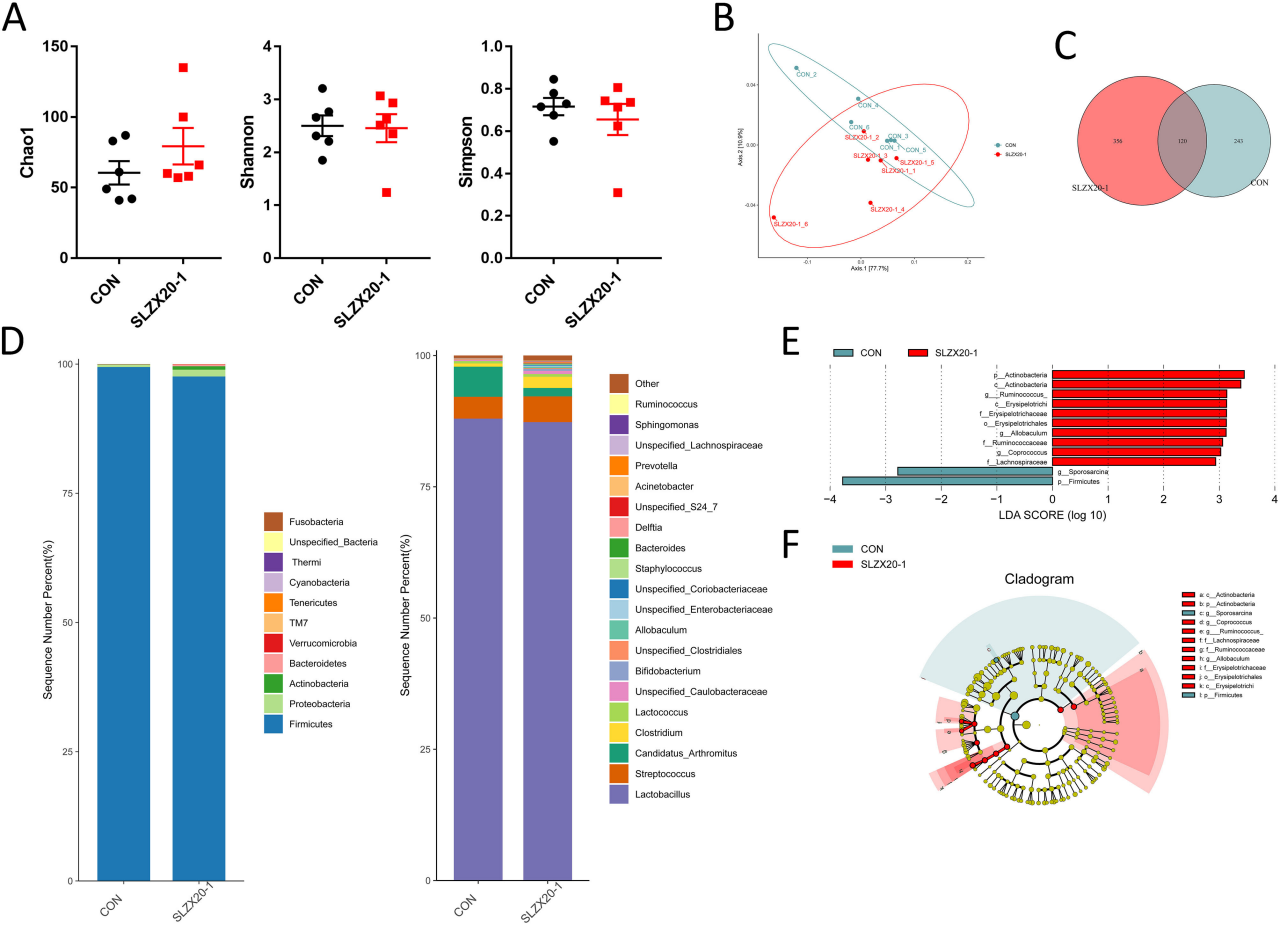
Effects of *L. amylovorus* SLZX20-1 microbial composition of the ileum. **(A)** The α-diversity comparisons were analyzed by Chao1, Shannon's diversity, and Simpson index, data were shown as mean ± SEM. **(B)** The β-diversity comparisons were analyzed by weighted UniFrac PCoA. **(C)** Common species analysis was shown by the Venn diagram. **(D)** Community composition of the gut microbiota at the phylum and genus levels. **(E)** Bacterial taxa differentially were identified by LEFSe using an LDA score threshold of >2.0 and *p* < 0.05. **(F)** LEfSe cladogram of microbial composition in ileum of control and *L. amylovorus* SLZX20-1-treated mice (LDA score >2, *p* < 0.05), red and gray nodes/shades indicate taxa that are significantly higher in relative abundance. The diameter of each node is proportional to the taxon's abundance, CON, and SLZX20-1 mean control and *L. amylovorus* SLZX20-1 (1 × 10^9^ CFU/ml) -treated mice, respectively.

In order to evaluate the effect of the strain on the intestinal microbial composition of mice, the ileum samples were analyzed by 16S rDNA sequencing, it can be seen that there is no significant difference between the *L. amylovorus* SLZX20-1 group (1 × 10^9^ CFU/ml SLZX20-1) and the control group in terms of α-diversity (Figure 5A) and β-diversity (Figure 5B) (*p* > 0.05), but the samples in the SLZX20-1 group are more dispersed in terms of β-diversity. According to the common strains analyzed by the Venn diagram, there were 120 common strains between the SLZX20-1 group and the control group, and 356 differential strains in the SLZX20-1 group (Figure 5C).

The taxonomic distributions were further assessed to find the most abundant bacterial OTUs in each sample region. Based on the bacterial relative impairment of the top 11 phyla, whether in the control group or the SLZX20-1 group, Firmicutes were the most abundant flora in the ileum (99.43 and 97.60%, respectively), followed by Proteobacteria (0.44 and 1.31%, respectively). In addition, the abundance of Actinobacteria (0.66%), Bacteroidetes (0.36%), Verrucomicrobia (0.04%), and TM7 (0.02%) in SLZX20-1 group was higher than that in the control group (0.05, 0.07, 0.02, 0.03%, respectively; Figure 5D, Supplementary Table 5).

At the genus level, a total of 20 genera were identified, *Lactobacillus* was the most prevalent, and the relative abundance was 87.98 and 87.31% in the control group and the SLZX20-1 group, respectively. A decreased proportion of *Candida_arthritis* was observed in SLZX20-1 group compared with the control group (from 5.73 to 1.61%), and an increased abundance of *Clostridium* was observed in the SLZX20-1 group compared with the control group (from 0.62 to 2.15%). The abundance of *Unspecified_Caulobacteraceae* in the SLZX20-1 group (0.60%) was higher than the control group (0.18%) (Figure 5D, Supplementary Table 6). Furthermore, it was found that the average distribution index of 11 genera among the top 20 genera in the SLZX20-1 group was higher than the control group, except two genera in the ileum (the *Unspecified_Coriobacteriaceae* 0.05% and *Prevotella* 0.02%).

To identify the bacterial species' most characteristics of the SLZX20-1 group, LEfSe analysis of the taxa was conducted with linear discriminant analysis (LDA) scores >2 and *p* < 0.05. This approach revealed that 12 OTUs were differentially present between the SLZX20-1 group and the control group (Figure 5E, Supplementary Table 7) and further visualized the result of the Kruskal-Wallis rank-sum test with a LEfSe cladogram (Figure 5F).

### Effects of *L. amylovorus* SLZX20-1 Microbial Composition of Colon

**Figure 6:**
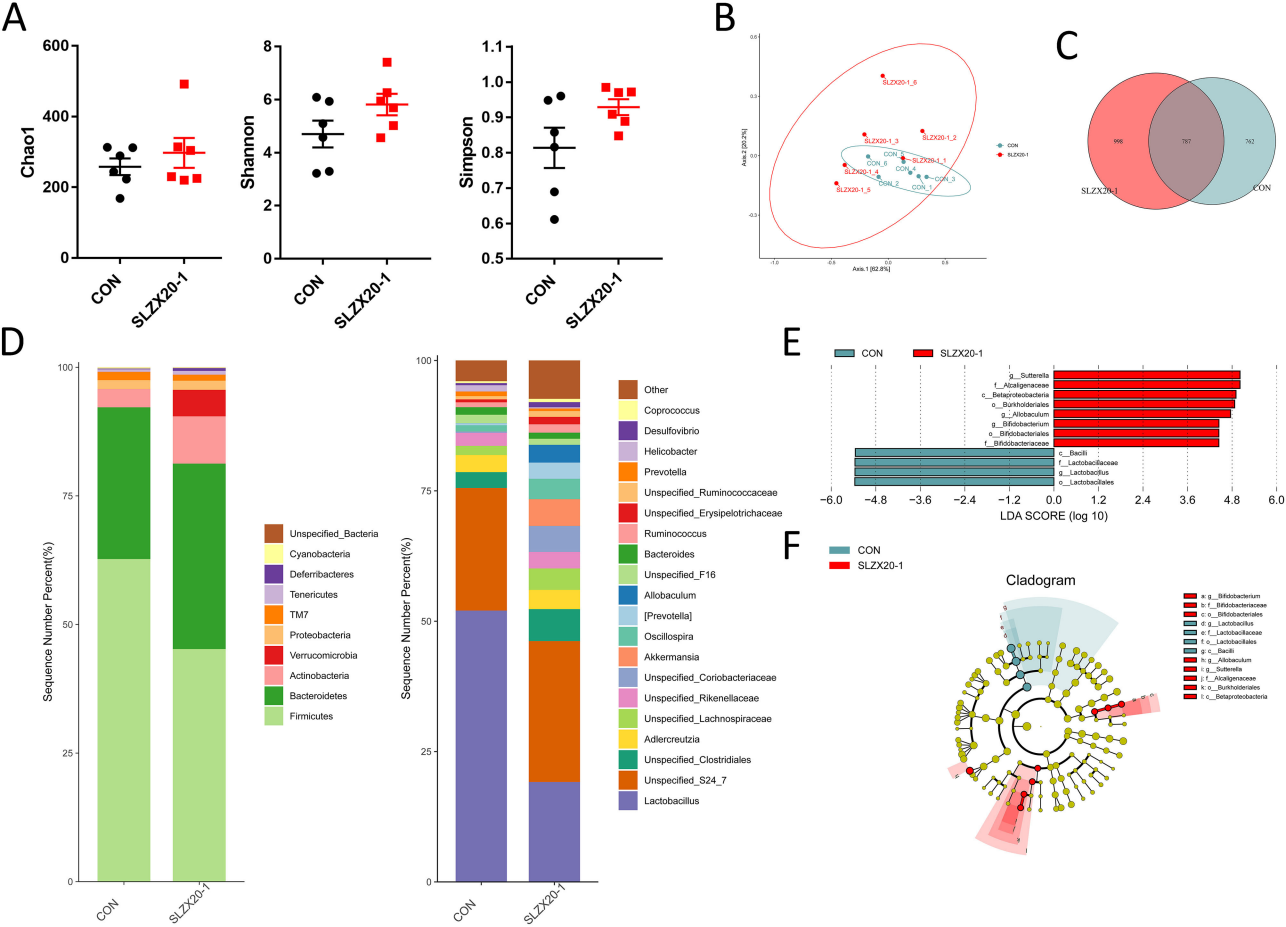
Effects of *L. amylovorus* SLZX20-1 microbial composition of colon. **(A)** The α-diversity comparisons were analyzed by Chao1, Shannon's diversity, and Simpson index, data were shown as mean ± SEM. **(B)** The β-diversity comparisons were analyzed by weighted UniFrac PCoA. **(C)** Common species analysis was shown by the Venn diagram. **(D)** Community composition of the gut microbiota at the phylum and genus levels. **(E)** Bacterial taxa differentially were identified by LEFSe using an LDA score threshold of >2.0 and *p* < 0.05. **(F)** LEfSe cladogram of microbial composition in colon of control and *L. amylovorus* SLZX20-1-treated mice (LDA score >2, *p* < 0.05), red and gray nodes/shades indicate taxa that are significantly higher in relative abundance. The diameter of each node is proportional to the taxon's abundance, CON, and SLZX20-1 mean control and *L. amylovorus* SLZX20-1 (1 × 10^9^ CFU/ml) -treated mice, respectively.

The colon microbial composition analysis is further explored in Figure 6, the result showed that the treatment of *L. amylovorus* SLZX20-1 resulted in a slight increase in Chao1, Shannon, and Simpson index, but they did not reach the significant level (Figure 6A) (*p* > 0.05). In β-diversity analysis, the samples in the SLZX20-1 group were more dispersed, which was similar to the ileum (Figure 5B). Venn diagram showed that there are 787 common strains between the SLZX20-1 group and the control group in the colon, and 998 differential strains in the SLZX20-1 group (Figure 6C).

At the phylum level, a total of 10 phyla were identified, the most abundant phyla in the colon of the control group and SLZX20-1 group is Firmicutes (62.72 and 45.22%, respectively), followed by Bacteroidetes (29.50 and 36.05%). In addition, the abundance of Actinobacteria (9.21%), Verrucomicrobia (5.15%), Tenericutes (0.73%), and Deferribacteres (0.55%) in the SLZX20-1 group was higher than the control group (3.55, 0.01, 0.47, and 0.15% in the control group, respectively) (Figure 6D, Supplementary Table 8).

The genus level was further analyzed, and the results showed that a total of 20 genera were identified. The dominant genus in the control group was *Lactobacillus* (52.04%), but in SLZX20-1 group, the dominant genus was changed to *Unspecified_S24_7* (27.03%) but not *Lactobacillus* (19.16%). In addition, the treatment of SLZX20-1 group increased the abundant of *Unspecified_Clostridiales* (6.13%), *Akkermansia* (5.15%), *Unspecified_Lachnospiraceae* (4.10%), *Oscillospira* (3.92%), *Allobaculum* (3.42%), and *[Prevotella]* (3.09%) than control group (3.04, 0.01, 1.74, 1.22, 0.02, and 0.36% in control group respectively). The abundance of *Prevotella* (0.51%) and *Helicobacter* (0.23%) was decreased in the SLZX20-1 group as compared with the control group (0.92 and 1.19% in the control respectively) (Figure 6D, Supplementary Table 9).

LEfSe analysis of the taxa showed that 12 OTUs were differentially present between the SLZX20-1 group and control group in the colon (Figure 6E, Supplementary Table 10), and the result of the Kruskal-Wallis rank-sum test was further visualized with a LEfSe cladogram (Figure 6F).

## Discussion

Probiotics have been shown to be beneficial to maintain healthy piglets, promote their growth, and improve feed conversion rate. Many microbes have been used as probiotics, but lactic acid bacteria appear to be the most potential probiotic candidate in pig nutrition (21). Early studies have isolated and screened lactic acid bacteria for probiotics for the preparation of pigs, indicating the existence of species specificity of probiotics (22). Compared with microorganisms from other species, the natural flora from the gut is easy to reproduce rapidly and reach a stable state in the gut, which indicate that it may play a more effective role in maintaining the balance between beneficial and harmful bacteria and promoting the health of the host (21). In our previous findings, we found that *L. amylovorus* is the dominant species in feces of weaned piglets, after further selection through an optional medium, we successfully isolated this *L. amylovorus* strain from Tibetan piglets, after the identification by 16S rDNA, we further named this strain as *L. amylovorus* SLZX20-1.

*Lactobacillus amylovorus* is a kind of lactic acid bacteria that can hydrolyze starch. It was first found and isolated from the mixed fermentation of cattle waste and corn by Nakamura (23). The colony is white, convex, smooth, round, with neat and opaque edges. It belongs to Gram-positive bacteria. The optimum pH is 5.5–6.0, but the pH 3.0–4.5 can still survive (24). Padmavathi et al. isolated a *Lactobacillus* strain with acid resistance, bile salt resistance, and degradable starch from soil and verified it with modified MRS medium and starch (25). These characteristics were also similar to the strains we isolated. Combined with the sequencing results, the isolated strain *L. amylovorus* SLZX20-1 was confirmed belonging to *L. amylovorus*.

The enzyme profile of *L. amylovorus* SLZX20-1 was detected by API-ZYM, and it was found that it could produce many enzymes. Aminopeptidase plays an important role in the hydrolysis of bitter peptides and the release of amino acids, which may be involved in flavor formation (26). The active α-galactosidase can hydrolyze α-galactosidase, which is a common anti-nutritional factor in legumes and affects nutrient Substances. α-glucosidase can catalyze the hydrolysis of α-1, 4-glucosidase, and decompose oligosaccharides, such as maltose and sucrose in the small intestine. β-glucosidase is a major component of cellulase, which hydrolyzes the cellulose disaccharide produced by cellulose degradation of endocellulase and exocellulase. It is noteworthy that β-glucosidase plays an important role in the bioconversion of olivin to hydroxytyrosol, which is an ideal food antioxidant (27). In addition, studies have found that *L. amylovorus* has the function of producing ferulate esterase (28). Ferulate esterase can improve the hydrolysis efficiency of lignin, cellulose, and hemicellulose in plant cell walls and release the antioxidant ferulic acid with antioxidant, bacteriostatic, and anti-inflammatory functions. In addition, α-chymotrypsin β-glucuronidase and N-acetyl-β-glucosaminase are related to intestinal diseases (29). However, *L. amylovorus* SLZX20-1 does not produce these enzymes, which can better explain its safety.

Studies have shown that *L. amylovorus* carrying S-layer protein isolated from the small intestine of piglets shows potential health-promoting effects both *in vitro* trial and in weaned piglets (30, 31). Therefore, this symbiotic strain may have potential as a probiotic feed additive during weaning. The primary condition for lactic acid bacteria to function as probiotics is to survive in the upper digestive tract and function in the intestinal environment. Therefore, acid and bile tolerance are the primary characteristics of probiotics in the intestinal environment. *L. amylovorus* SLZX20-1 has good tolerance to the acidic environment and a certain tolerance to low bile salt concentration. The pH value of *L. amylovorus* SLZX20-1 fermentation broth cultured for 24 h can be lower than 4 h, showing a strong acid production ability. By ion chromatography analysis, the supernatant mainly consisted of lactic acid and acetic acid, of which lactic acid content was the highest.

Growth inhibition of pathogens is one of the most direct and important ways for probiotics to antagonize pathogens. Considered the most fundamental property of probiotic strains, many bacteria are credited with probiotic properties *in vitro* showed on the common inhibitory activity for pathogenic bacteria, such as *E. coli, Listeria*, and *Salmonella* (31). In fact, many probiotics can produce antibacterial material, lactic acid, acetic acid, and aromatic compounds, such as hydrogen peroxide secondary metabolites, these substances also can inhibit the growth of pathogens, such as *Sramana, E. coli, Clostridium*, and other harmful organisms (32). In the present study, *L. amylovorus* SLZX20-1 showed strong ability in inhibiting the growth of pathogenic bacteria, and the supernatant of *L. amylovorus* SLZX20-1 had an inhibitory effect on *E. coli* and *Salmonella* while not the heavy suspension liquid, explain the antibacterial substances were associated with bacterial metabolites, rather than the bacteria itself in this study, and may be associated with lactic acid, low pH value and antibacterial compounds. It is important to note that although the chosen indicator bacteria showed inhibitory function, however, its inhibition zone disappeared when the upper clearance was adjusted to neutral and reappeared when the pH value was 4. This result was consistent with previous research results, and the acidic environment caused by lactic acid is a factor that may affect the survival of pathogens (33). In addition to the antibacterial effect due to the pH reduction, lactic acid also acts as an outer membrane penetrant of Gram-negative bacteria and may act as an enhancer of other antibacterial substances, inducing bacteria to exert antibacterial capacity (34), and chelating essential nutrients, such as iron ions (35).

In the present study, *L. amylovorus* SLZX20-1 has certain adhesion abilities to porcine intestinal epithelial cells, IPEC-J2, but it has not been identified whether S layer protein is involved in its adhesion to host cells. Some studies have found that S layer protein on the surface of *L. amylovorus* is not directly involved in its adhesion to intestinal epithelial cells (33). The adhesion ability of *L. amylophilus* isolated from silage was significantly worse than that isolated from porcine intestinal epithelial cells (33). Anti-adhesion strategies can effectively inhibit mucosal surface pathogen-mediated disease, especially in combination with selective pressure of competitive exclusion by members of the normal intestinal flora (36). In present study, co-culture was used to investigate whether *L. amylovorus* SLZX20-1 could inhibit the adhesion ability of pathogenic bacteria to intestinal epithelial cells by competitive exclusion replacement in three ways. Studies have confirmed that *L. amylovorus* can inhibit the adhesion ability of pathogenic bacteria to intestinal epithelial cells by competitive exclusion replacement. *L. amylovorus* DSM 16698 was adhesion to IPEC-1 cells and its competitive inhibition of ETEC binding to IPEC-1 cells (37). However, in addition to competitive inhibition, there may also be other mechanisms, such as secretion of inhibitory factors, because strains with poor adhesion can also inhibit ETEC adhesion (31). Contrary to the above results, *L. amylovorus* SLZX20-1 isolated in this study could not inhibit ETEC pairs by means of competitive exclusion replacement the adhesion effect of IPEC-J2, this may be due to the insufficient dose of *L. amylovorus* SLZX20-1, which produces less acid not enough to affect the growth of pathogenic bacteria, and this speculation needs further exploration.

Host defense peptides are mainly produced by intestinal epithelial cells and gastrointestinal phagocytes. They are an important part of the innate immune system and play an important role in pathogen clearance (38). HDPs are usually studied because of their antibacterial properties and have been proven to kill bacteria, viruses, fungi, protozoa, and even cancer cells (31). Different probiotic strains show different abilities to induce HDP production (39). Recent studies have shown that lactic acid bacteria can stimulate the expression of HDPs while not inducing inflammatory response (40) and enhance the synthesis of endogenous HDPs, which is conducive to the early response to infection and inflammation (41). We observed that different concentrations of *L. amylovorus* SLZX20-1 showed different effects on the expression of HDPs, in general, high dose and medium dose showed significant upregulation of *NK-lysin, PEP2C, PBD-1*, and *PG1-5*. Moreover, the ability of probiotics to regulate HDPs production varies from strain to strain, this may lead to different effects of different strains (39). Previous studies have proved that *L. amylovorus* and *Lactobacillus reuteri* also have the ability to upregulate HDP expression but not cause inflammation (40, 42) in addition to regulating defense response, HDPs are also related to nutrient digestibility, intestinal morphology, and growth performance of weaned piglets (43), these findings indicate that the expression of HDP gene induced by *L. amylovorus* SLZX20-1 is not only conducive to innate immune response but also conducive to body health and production performance.

*L. amylovorus* can utilize starch is one of the reasons for its extensive research. Tavea et al. isolated a strain of *L. amylovorus* from soil, which can secrete highly thermally stable α-amylase (44). Bertrand et al. enhanced the understanding of α-amylase production of *Bacillus amyloliquefaciens* 04BBA15 and *Lactobacillus fermentum* 04BBA19 through microbial interaction (45). In silage, inoculating *L. amylovoru*s can significantly increase the soluble carbohydrate content of silage (46). We observed that *L. amylovoru*s supplementation significantly increased feed intake in mice. Lee et al. proposed that starch digestion is related to the expression of mechanisms related to appetite control and that fermentation in the large intestine occurs when starch and/or dietary fiber escape the stomach and small intestine digestion. Undigested and fermentable carbohydrates arriving at the large intestine are fermented to produce SCFAs. These SCFAs are chemosensory by FFAR2 receptors in the colon, leading to the secretion of appetite-suppressing intestinal peptide (PYY), thereby reducing feed intake (47). Therefore, we can speculate that the increase of feed intake in mice may be related to the digestion and absorption of starch and other carbohydrates in the ileum and colon by *L. amylovorus* SLZX20-1.

Among the changes in gut microbiota after the treatment of *L. amylovorus* SLZX20-1, the most striking finding is that, at the genus level, the dominant genus in the colon was replaced by *S24-7* from *Lactobacillus*. In addition, we also observed similar abundance changes in the ileum of *Lactobacillus* and *S24-7* on genus level (Supplementary Table 6). *S24-7* family (belongs to phylum *Bacteroidetes*) has recently been named as *Muribaculaceae* and showed prominent complex carbohydrates degradation (48), *S24-7* were decreased in the feeding experiment of high-calorie or carbohydrate-enriched diets (49–51), further, the composition of carbohydrates in intestine is an important factor to gut microbiota (52), we speculated that the changes in the abundance of *S24-7* may be related to the composition of carbohydrates in the intestinal tract. After the oral treatment, *L. amylovorus* SLZX20-1 entered the intestine and adhered to intestinal epithelial cells, *L. amylovorus* SLZX20-1 may become potential competitors of *Lactobacillus* in the utilization of carbohydrate, further resulting in the decrease of *Lactobacillus*, on the other hand, the ability of complex carbohydrates degradation may help *S24-7* fit the altered composition of carbohydrates and increase their abundance. Up to now, the mechanism of starch metabolism and preferential utilization of different carbohydrates by *L. amylovorus* are still not unclear, in addition, the location of *L. amylovorus* colonization in intestine has not been determined, therefore, whether *L. amylovorus* affected gut microbiota by changing the composition of intestinal carbohydrates needs further study. In the present study, *L. amylovorus* significantly increased the feed intake of mice, but there was no significant increase in body weight. *L. amylovorus* has been proved to having protective effects against diet-induced obesity *in vivo* (53). In addition, studies on obesity have confirmed that the abundance of *Akkermansia* (54) and *S24-7* (55) is significantly negatively correlated with obesity. We also found that *L. amylovorus* increased the abundance of *Akkermansia* and *S24-7* in the present study, which indicated a correlation may exist among these findings. Therefore, the mechanism of *L. amylovorus* regulating intestinal flora, eating, and obesity still needs to be further studied.

## Conclusion

The novel strain isolated from Tibetan piglets was named *L. amylovorus* SLZX20-1, it can secrete a variety of bioactive enzymes, such as galactosidase, glucosidase, and ferulic acid esterase to eliminate antinutritional factors or decompose oligosaccharides. In addition, the main function of this strain is to produce a large number of SCFAs for antibacterial function, adhere to intestinal epithelial cells, and improve the expression of HDPs in intestinal epithelial cells. At present, a prominent problem of *L. amylovorus* is its unclear priority utilization sequence of different carbon sources, which limits the further development of *L. amylovorus*. In the present study, *L. amylovorus* SLZX20-1 changed the gut microbiota composition, especially the abundance of *S24-7* and *Lactobacillus* in the colon. These genera were significantly different in carbohydrate utilization, and it would be interesting to see if this was related to the role of *L. amylovorus in vivo*, meanwhile, enhanced HDPs expression by interaction with intestinal epithelial cells may also be a potential factor for microbial changes in the host intestinal flora. Therefore, as potential new food and feed additives, *L. amylovorus* SLZX20-1 showed good results in antibacterial activity, acid productivity, and well interaction with the host. After further exploration of its safety evaluation *in vivo* and underlying mechanisms of its effects on gut microbiota, *L. amylovorus* SLZX20-1 would be applied as a novel probiotic.

## Data Availability Statement

The datasets presented in this study can be found in online repositories. The names of the repository/repositories and accession number(s) can be found below: https://www.ncbi.nlm.nih.gov/PRJNA792839.

## Ethics Statement

The animal study was reviewed and approved by Animal Protection and Utilization Organization Committee of China Agricultural University.

## Author Contributions

The research was mainly conceived and designed by XM and conducted by YZ, JS, and JZ. YZ analyzed the data. JS and JZ wrote the manuscript. YZ, ZL, and LJ contributed to sample collection and analysis. XM critically reviewed the manuscript. All authors read and approved the final manuscript.

## Funding

This work was supported by the National Natural Science Foundation of China (31930106, 31829004, and 31722054), the National 10-thousand Talents Program of China (23070201), and the 111 Project (B16044).

## Conflict of Interest

XM, YZ, and ZL hold a Chinese patent for the protective attributes of the microbial strain reported in this study: Patent number CN113621538A, “A *Lactobacillus amylophilus* and its application.” The remaining authors declare that the research was conducted in the absence of any commercial or financial relationships that could be construed as a potential conflict of interest.

## Publisher's Note

All claims expressed in this article are solely those of the authors and do not necessarily represent those of their affiliated organizations, or those of the publisher, the editors and the reviewers. Any product that may be evaluated in this article, or claim that may be made by its manufacturer, is not guaranteed or endorsed by the publisher.

## Abbreviations

ADFI: average feed intake
ADG: average daily gain
CFU: colony forming units
*C. rodentium*: *Citrobacter rodentium*
E. coli: Escherichia coli
*ETEC*: *Enterotoxigenic E. coli*
F:G: feed consumption to gain ratio
HDPs: host defense peptides
IPEC-J2: intestinal porcine epithelial cells
*L. amylovorus*: *Lactobacillus amylovorus*
SCFAs: short-chain fatty acids
*S. aureus*: *Staphylococcus aureus*
*S. pullorum*: *Salmonella pullorum*
*S. typhimurium*: *Salmonella typhimurium*.
