# Supplementary material for: Tibetan Pig-Derived Probiotic Lactobacillus amylovorus SLZX20-1 Improved Intestinal Function via Producing Enzymes and Regulating Intestinal Microflora
Source: Front Nutr. 2022 Mar 29;9:846991. doi: 10.3389/fnut.2022.846991 (PMC9002122; doi:10.3389/fnut.2022.846991)
Supplement: Supplementary file 1 [file Data_Sheet_1.docx]

**Supplementary materials**

**Supplementary Figure 1** The amplification results of *L. amylovorus* SLZX20-1 by PCR


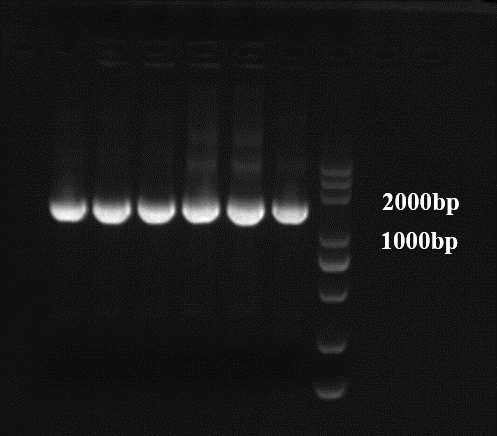


**Supplementary Figure 2** The utilization of starch by *L. amylovorus* SLZX20-1. The red circle represent the area that are not stained by iodine solution


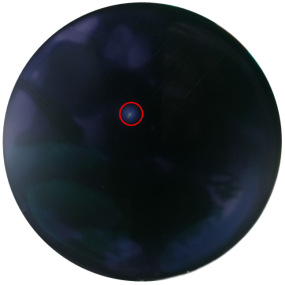


**Supplementary Figure 3.** Antibacterial activity of *L. amylovorus* SLZX20-1. The inhibitory effect of *L.amylovorus* SLZX20-1 against common pathogenic bacteria, Hole 1 is the positive control group (doxycycline), hole 2 is the group of sediment of *L. amylovorus* SLZX20-1, hole 3 is the negative control group, hole 4 is group of the fermentation broth of *L. amylovorus* SLZX20-1, hole 5 is group of bacterial suspension of *L. amylovorus* SLZX20-1


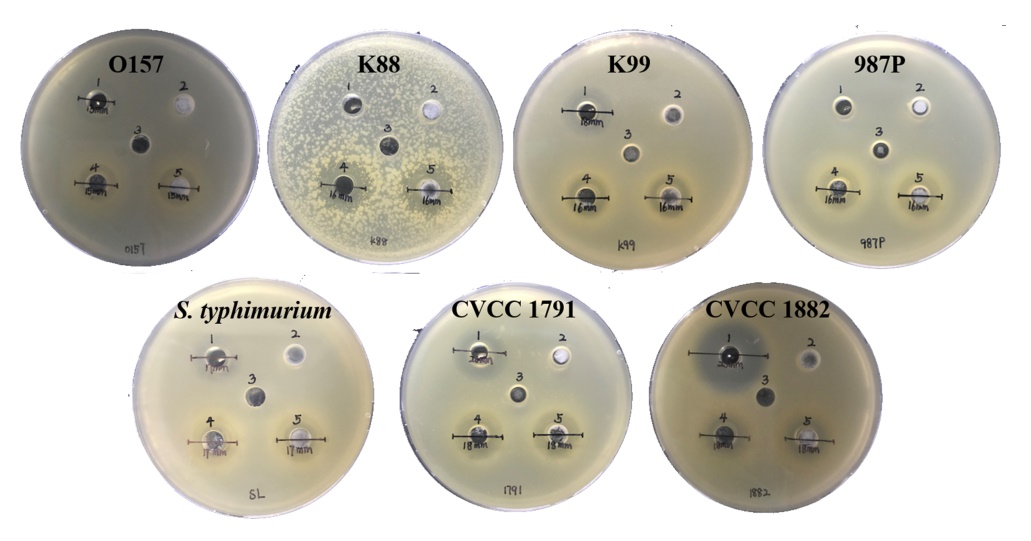


**Supplementary Figure 4** The effects of feed-supplementation with *L. amylovrous* SLZX20-1 on intestinal morphology of mice. Representative pictures of different intestinal segments were shown, bar=100 μm


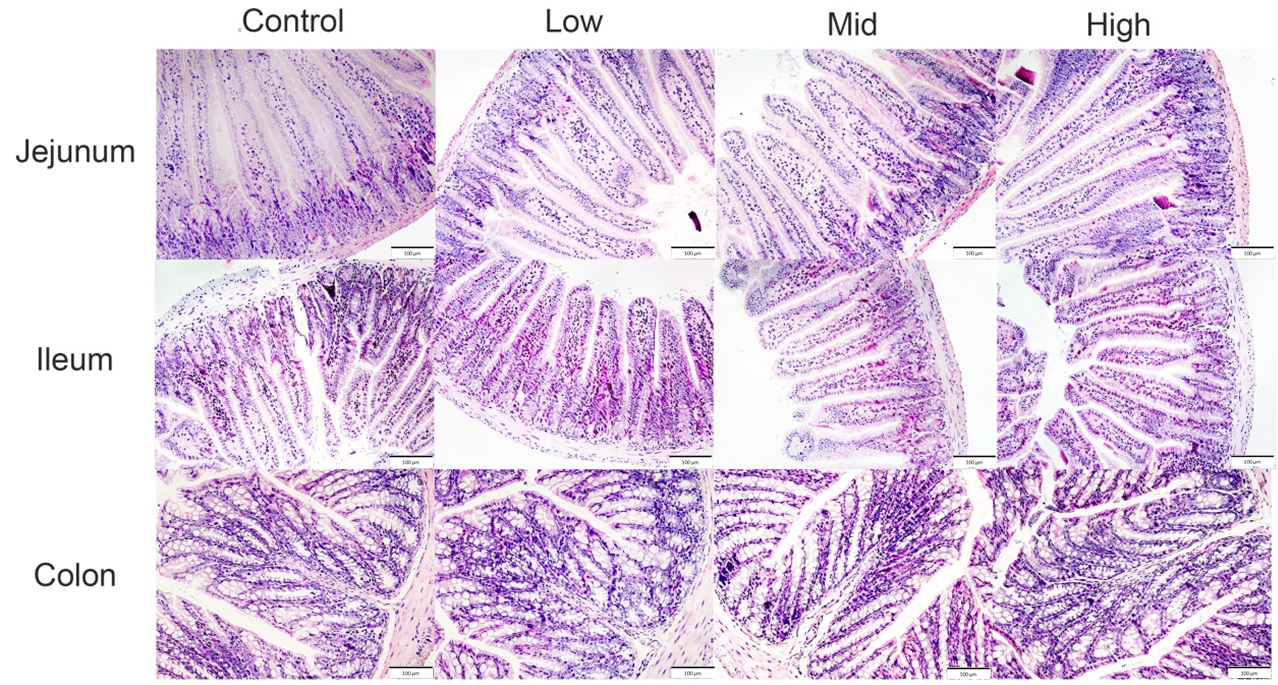


**Supplementary Table 1** Primer sequences of target genes used for qRT-PCR assays

| Gene name | Primer sequence (5’ to 3’) | Tm (℃) | Product size (bp) |
| --- | --- | --- | --- |
| GAPDH | F: CCCCTTCATTGACCTCCACT | 60 | 129 |
|  | R: TGGAAGATGGTGATGGCCTT |  |  |
| NK-lysin | F: GCCTCATCTGTGAGTCTTGTCG | 60 | 78 |
|  | R: CAGTGTCCTCGTTGGGTTGTG |  |  |
| PBD1 | F: CACCGCCTCCTCCTTGTATT | 60 | 176 |
|  | R: TTGCAGCATTTGACTTGGGG |  |  |
| PEP2C | F:AAGTCTCACCTGTTACGCCA | 60 | 112 |
|  | R:TGCCTTCACTTCTCTTGCAG |  |  |
| PG1-5 | F: GTAGGTTCTGCGTCTGTGTCG | 60 | 166 |
|  | R: CAAATCCTTCACCGTCTACCA |  |  |
| PR39 | F: CAAGGAGAACGGGCGAGTG | 60 | 159 |
|  | R: CTTGGTGGGAAAAACGGAGGT |  |  |

**Supplementary Table 2** The biochemical characteristics of *L. amylovorus* SLZX20-1

| Items | Results | Items | Results |
| --- | --- | --- | --- |
| Fibrodilose | +++ | Aescin | - |
| Maltose | +++ | Mannitol | - |
| Salicin | + | Sorbitol | - |
| Sucrose | + | 1% Sodium hippurate | - |
| Raffinose | + |  |  |
| Lactose | ++ |  |  |
| Synanthrin | + |  |  |

Note: “+” represents positive, which means it can ferment this carbohydrate; “++” represents it can ferment better; “+++” represents it has the best ferment effect; “-” represents negative, which means it can not ferment this carbohydrate.

**Supplementary Table 3** The enzyme activities results of *L. amylovorus* SLZX20-1

| Serial number | Enzyme | Results | Score |
| --- | --- | --- | --- |
| 1 | Control | - | - |
| 2 | Alkaline phosphatase | + | 2 |
| 3 | Esterase(C4) | + | 2 |
| 4 | Lipoesterase(C8) | - | - |
| 5 | Lipoidase(C14) | - | - |
| 6 | Leucine arylaminase | + | 5 |
| 7 | Valine arylaminase | + | 1 |
| 8 | Cystine arylaminase | + | 4 |
| 9 | Trypsin | + | 1 |
| 10 | Chymotrypsin | - | - |
| 11 | Acid phosphatase | + | 5 |
| 12 | Naphthol-AS-BI-phosphohydrola | + | 5 |
| 13 | α-galactosidase | + | 3 |
| 14 | β-galactosidase | + | 5 |
| 15 | β- glucuronidase | - | - |
| 16 | α-glucosidase | + | 5 |
| 17 | β-glucosidase | + | 4 |
| 18 | N-Acetylglucosaminase | - | - |
| 19 | α-mannosidase | - | - |
| 20 | β-fucosidase | - | - |

Note： A score ranging from 0-5 represents the color depth which suggest the response intensity. 0 corresponds to a negative reaction, 5 is the strongest reaction, 2 to 4 are situated between the two points

**Supplementary Table 4** The inhibitory results of *L. amylovorus* SLZX20-1 on the adhesion of *E.coli* K88

| Items | Log CFU of adhesive *E. coli* K88 | | |
| --- | --- | --- | --- |
|  | *E. coli* K88 | *E. coli* K88 + *L. amylovorus* SLZX20-1 | P value |
| Compete | 7.91 ± 0.05 | 7.95 ± 0.03 | 0.42 |
| Exclude | 8.32 ± 0.04 | 8.24 ± 0.46 | 0.29 |
| Replace | 8.03 ± 0.25 | 8.01 ± 0.03 | 0.52 |

**Supplementary Table 5** Community composition of the gut microbiota in ileum at phylum level

|  | CON | SLZX20-1 |
| --- | --- | --- |
| Firmicutes | 0.994299704 | 0.976035105 |
| Proteobacteria | 0.00441619 | 0.013107036 |
| Actinobacteria | 0.000481104 | 0.006600445 |
| Bacteroidetes | 0.000739889 | 0.003562369 |
| Verrucomicrobia | 1.99E-05 | 0.000370292 |
| TM7 | 2.86E-05 | 0.000170556 |
| Tenericutes | 5.81E-06 | 7.00E-05 |
| Cyanobacteria | 8.72E-06 | 4.17E-05 |
| Thermi | 0 | 1.90E-05 |
| Unspecified_Bacteria | 0 | 1.56E-05 |
| Fusobacteria | 0 | 7.91E-06 |

**Supplementary Table 6** Community composition of the gut microbiota in ileum at genus level

|  | CON | SLZX20-1 |
| --- | --- | --- |
| *Lactobacillus* | 0.879818183 | 0.8730914 |
| *Streptococcus* | 0.041674495 | 0.0491374 |
| *Candidatus_Arthromitus* | 0.057312146 | 0.0160634 |
| *Clostridium* | 0.006157015 | 0.0214618 |
| *Lactococcus* | 0.004600978 | 0.0051564 |
| *Unspecified_Caulobacteraceae* | 0.001765284 | 0.0059967 |
| *Bifidobacterium* | 0.000120114 | 0.0036254 |
| *Unspecified_Clostridiales* | 0.001575284 | 0.0015783 |
| *Allobaculum* | 5.15E-05 | 0.0027049 |
| *Unspecified_Enterobacteriaceae* | 0.00058913 | 0.0020221 |
| *Unspecified_Coriobacteriaceae* | 3.50E-05 | 0.0016127 |
| *Staphylococcus* | 0.000401473 | 0.0010674 |
| *Bacteroides* | 0.000197627 | 0.0011404 |
| *Delftia* | 0.000426701 | 0.0008747 |
| *Unspecified_S24_7* | 0.000329353 | 0.0009408 |
| *Acinetobacter* | 0.000274097 | 0.0009017 |
| *Prevotella* | 2.12E-05 | 0.0008831 |
| *Unspecified_Lachnospiraceae* | 0.00012012 | 0.0007779 |
| *Sphingomonas* | 0.000172179 | 0.0006975 |
| *Ruminococcus* | 5.90E-05 | 0.0007042 |
| *Other* | 0.004299055 | 0.0095617 |

**Supplementary Table 7** The analysis results of the bacterial taxa differentially in mice ileum by LEFSe

|  | **Mean relative abundance within the group**  **(Logarithmic conversion value)** | **Location** | **LDA score** | **P value** |
| --- | --- | --- | --- | --- |
| ***k__Bacteria.p__Firmicutes.c__Bacilli.o__Bacillales.f__Planococcaceae.g__Sporosarcina*** | **2.715172163** | **CON** | **2.781728** | **0.02223** |
| ***k__Bacteria.p__Firmicutes.c__Erysipelotrichi.o__Erysipelotrichales*** | **3.443173158** | **SLZX20-1** | **3.126298** | **0.030889** |
| ***k__Bacteria.p__Firmicutes.c__Clostridia.o__Clostridiales.f__Ruminococcaceae*** | **3.143672489** | **SLZX20-1** | **3.0588** | **0.024975** |
| ***k__Bacteria.p__Firmicutes.c__Clostridia.o__Clostridiales.f__Lachnospiraceae.g___Ruminococcus_*** | **2.372936382** | **SLZX20-1** | **3.133946** | **0.004763** |
| ***k__Bacteria.p__Actinobacteria.c__Actinobacteria*** | **3.621835413** | **SLZX20-1** | **3.386562** | **0.006485** |
| ***k__Bacteria.p__Firmicutes.c__Erysipelotrichi.o__Erysipelotrichales.f__Erysipelotrichaceae.g__Allobaculum*** | **3.438328294** | **SLZX20-1** | **3.121605** | **0.030889** |
| ***k__Bacteria.p__Actinobacteria*** | **3.6873388** | **SLZX20-1** | **3.448896** | **0.003948** |
| ***k__Bacteria.p__Firmicutes.c__Erysipelotrichi.o__Erysipelotrichales.f__Erysipelotrichaceae*** | **3.443173158** | **SLZX20-1** | **3.129066** | **0.030889** |
| ***k__Bacteria.p__Firmicutes*** | **5.998851315** | **CON** | **3.772663** | **0.016309** |
| ***k__Bacteria.p__Firmicutes.c__Clostridia.o__Clostridiales.f__Lachnospiraceae*** | **3.202923044** | **SLZX20-1** | **2.934815** | **0.037041** |
| ***k__Bacteria.p__Firmicutes.c__Clostridia.o__Clostridiales.f__Lachnospiraceae.g__Coprococcus*** | **2.57410584** | **SLZX20-1** | **3.025189** | **0.007397** |
| ***k__Bacteria.p__Firmicutes.c__Erysipelotrichi*** | **3.443173158** | **SLZX20-1** | **3.13106** | **0.030889** |

**Supplementary Table 8** Community composition of the gut microbiota in colon at phylum level

|  | CON | SLZX20-1 |
| --- | --- | --- |
| Firmicutes | 0.627223 | 0.452236 |
| Bacteroidetes | 0.294993 | 0.360451 |
| Actinobacteria | 0.035478 | 0.092058 |
| Verrucomicrobia | 0.000117 | 0.051505 |
| Proteobacteria | 0.017217 | 0.017731 |
| TM7 | 0.016267 | 0.011752 |
| Tenericutes | 0.004748 | 0.007312 |
| Deferribacteres | 0.001466 | 0.005487 |
| Cyanobacteria | 0.002376 | 0.001267 |
| Unspecified_Bacteria | 0.000115 | 0.0002 |

**Supplementary Table 9** Community composition of the gut microbiota in colon at genus level

|  | CON | SLZX20-1 |
| --- | --- | --- |
| *Lactobacillus* | 0.520381 | 0.191608 |
| *Unspecified_S24_7* | 0.235159 | 0.270277 |
| *Unspecified_Clostridiales* | 0.0304 | 0.061286 |
| *Adlercreutzia* | 0.033017 | 0.037063 |
| *Unspecified_Lachnospiraceae* | 0.017441 | 0.040978 |
| *Unspecified_Rikenellaceae* | 0.024884 | 0.031689 |
| *Unspecified_Coriobacteriaceae* | 0.002398 | 0.04974 |
| *Akkermansia* | 0.000117 | 0.051505 |
| *Oscillospira* | 0.012186 | 0.03917 |
| *[Prevotella]* | 0.003569 | 0.030908 |
| *Allobaculum* | 0.000238 | 0.034168 |
| *Unspecified_F16* | 0.016267 | 0.01165 |
| *Bacteroides* | 0.014422 | 0.011596 |
| *Ruminococcus* | 0.00935 | 0.01608 |
| *Unspecified_Erysipelotrichaceae* | 0.005703 | 0.014308 |
| *Unspecified_Ruminococcaceae* | 0.006069 | 0.01096 |
| *Prevotella* | 0.009152 | 0.005069 |
| *Helicobacter* | 0.011878 | 0.002293 |
| *Desulfovibrio* | 0.004057 | 0.009785 |
| *Coprococcus* | 0.003659 | 0.00646 |
| *Other* | 0.039654 | 0.073408 |

**Supplementary Table 10** The analysis results of the bacterial taxa differentially in mice colon by LEFSe

|  | **Mean relative abundance within the group**  **(Logarithmic conversion value)** | **Location** | **LDA score** | **P value** |
| --- | --- | --- | --- | --- |
| ***k__Bacteria.p__Actinobacteria.c__Actinobacteria.o__Bifidobacteriales.f__Bifidobacteriaceae.g__Bifidobacterium*** | **4.005899** | **SLZX20-1** | **4.444358** | **0.04951** |
| ***k__Bacteria.p__Firmicutes.c__Bacilli.o__Lactobacillales.f__Lactobacillaceae.g__Lactobacillus*** | **5.884779** | **CON** | **5.359186** | **0.024975** |
| ***k__Bacteria.p__Proteobacteria.c__Betaproteobacteria.o__Burkholderiales.f__Alcaligenaceae*** | **3.881967** | **SLZX20-1** | **5.012668** | **0.024975** |
| ***k__Bacteria.p__Firmicutes.c__Bacilli.o__Lactobacillales*** | **5.893958** | **CON** | **5.363035** | **0.016309** |
| ***k__Bacteria.p__Firmicutes.c__Bacilli.o__Lactobacillales.f__Lactobacillaceae*** | **5.884779** | **CON** | **5.359186** | **0.024975** |
| ***k__Bacteria.p__Proteobacteria.c__Betaproteobacteria.o__Burkholderiales*** | **3.887026** | **SLZX20-1** | **4.867532** | **0.010406** |
| ***k__Bacteria.p__Proteobacteria.c__Betaproteobacteria*** | **3.889307** | **SLZX20-1** | **4.903876** | **0.010406** |
| ***k__Bacteria.p__Actinobacteria.c__Actinobacteria.o__Bifidobacteriales.f__Bifidobacteriaceae*** | **4.005899** | **SLZX20-1** | **4.441513** | **0.04951** |
| ***k__Bacteria.p__Actinobacteria.c__Actinobacteria.o__Bifidobacteriales*** | **4.005899** | **SLZX20-1** | **4.441592** | **0.04951** |
| ***k__Bacteria.p__Firmicutes.c__Bacilli*** | **5.89447** | **CON** | **5.348779** | **0.024975** |
| ***k__Bacteria.p__Proteobacteria.c__Betaproteobacteria.o__Burkholderiales.f__Alcaligenaceae.g__Sutterella*** | **3.881967** | **SLZX20-1** | **5.012668** | **0.024975** |
| ***k__Bacteria.p__Firmicutes.c__Erysipelotrichi.o__Erysipelotrichales.f__Erysipelotrichaceae.g__Allobaculum*** | **4.92404** | **SLZX20-1** | **4.758066** | **0.021025** |
